# CD44 increases the efficiency of distant metastasis of breast cancer

**DOI:** 10.18632/oncotarget.3410

**Published:** 2015-03-19

**Authors:** Suzanne McFarlane, Jonathan A. Coulter, Paul Tibbits, Anthony O'Grady, Cheryl McFarlane, Nicola Montgomery, Ashleigh Hill, Helen O. McCarthy, Leonie S. Young, Elaine W. Kay, Clare M. Isacke, David J.J. Waugh

**Affiliations:** ^1^ Centre for Cancer Research and Cell Biology, Queen's University Belfast, Belfast, Northern Ireland; ^2^ Royal College of Surgeons in Ireland, Beaumont Hospital, Dublin, Ireland; ^3^ Breakthrough Breast Cancer Research Centre, The Institute of Cancer Research, London, UK; ^4^ School of Pharmacy, Queen's University Belfast, Belfast, Northern Ireland

**Keywords:** CD44, metastasis, breast cancer

## Abstract

Metastasis is the predominant cause of death from cancer yet we have few biomarkers to predict patients at increased risk of metastasis and are unable to effectively treat disseminated disease. Analysis of 448 primary breast tumors determined that expression of the hylauronan receptor CD44 associated with high grade (*p* = 0.046), ER- (*p* = 0.001) and PR-negative tumors (*p* = 0.029), and correlated with increased distant recurrence and reduced disease-free survival in patients with lymph-node positive or large tumors. To determine its functional role in distant metastasis, CD44 was knocked-down in MDA-MB-231 cells using two independent shRNA sequences. Loss of CD44 attenuated tumor cell adhesion to endothelial cells and reduced cell invasion but did not affect proliferation *in vitro*. To verify the importance of CD44 to post-intravasation events, tumor formation was assessed by quantitative *in vivo* imaging and post-mortem tissue analysis following an intra-cardiac injection of transfected cells. CD44 knock-down increased survival and decreased overall tumor burden at multiple sites, including the skeleton *in vivo*. We conclude that elevated CD44 expression on tumour cells within the systemic circulation increases the efficiency of post-intravasation events and distant metastasis *in vivo*, consistent with its association with increased distant recurrence and reduced disease-free survival in patients.

## INTRODUCTION

CD44 is a cell-surface trans-membrane protein and is the principal receptor for hyaluronic acid (HA), a major component of the ECM. Moreover, the CD44^+^/CD24−^/low^ phenotype reputedly distinguishes tumorigenic from non-tumourigenic breast cancer cells [1, 2]. CD44^+^/CD24^−/low^ cells also exhibit enhanced invasive properties and favor distant metastasis [3, 4]. Furthermore CD44-expressing breast cancer cells with stem-like characteristics have been detected within the bone marrow of breast cancer patients presenting with early-stage disease [5].

As a result of differential splicing, CD44 is expressed as a series of standard (CD44s) or variant (CD44v) isoforms. Experimental studies in breast cancer models support that expression of standard and variant isoforms of CD44 increases disease-progressing and metastatic behavior. For example, tetracycline-regulated induction of CD44s in MCF-7 cells *in vivo* increases their dissemination of breast cancer cells to the liver [6] while CD44 enrichment has been detected in estrogen receptor (ER)-negative breast cancer cells that have disseminated to regional lymph nodes [7]. Furthermore, isoform-switching to promote expression of CD44s has been reported as being critical to epithelial-mesenchymal transition (EMT) in breast cancer models *in vivo*, promoted through the activation of Akt signalling [8]. However, there is a marked discordance between experimental and clinical data in the literature. For example, recent immunohistochemical studies report that CD44s expression correlates with improved overall survival in breast cancer patients, and specifically to increased survival in patients with lymph node involvement [9]. Earlier studies measuring mRNA and protein levels also report CD44s to be predictive of better outcome. As such, there is a continued need to develop a comprehensive understanding of the biochemistry, pharmacology and functional roles of CD44, in order to articulate the significance of this glycoprotein in disease.

Increased stromal HA production is also associated with poorly differentiated and infiltrating breast carcinomas, the spread of tumor cells to axillary lymph nodes and decreased survival [11, 12]. HA and CD44 co-localize in disseminated lesions within the lymph nodes, supporting a role for this receptor in mediating the HA-induced regional spread of breast cancer, consistent with recent immunohistochemical studies. With respect to potential roles in metastasis, HA is detected in the bone marrow sinusoidal endothelium and the lung microvasculature [13–15], two prominent sites of breast cancer metastasis. HA presentation on the endothelium is proposed to assist the homing of CD44-positive haematopoietic progenitor cells. Furthermore, high levels of circulating HA have recently been reported to inhibit metastasis, through a mechanism attributed to its role in preventing tumor cell interactions with the HA-displayed on the vascular endothelial cells [16].

Outgrowth of secondary tumors is initially dependent on the adhesion of circulating tumor cells to the endothelium lining the blood vessels within those tissues and their subsequent transmigration across the endothelial barrier. It is proposed that tumor cells adopt similar strategies as exploited by lymphocytes in undergoing extravasation [17, 18]. CD44 has a well-described role in mediating the adhesion and extravasation of T-lymphocytes in *in vitro* and *in vivo* models, initiating and underpinning a subsequent integrin receptor-promoted “firm” adhesion [19–22]. Similarly, we have previously shown that CD44 potentiates the adhesion of breast and prostate cancer cells to bone marrow endothelial cells (BMECs) *in vitro* [23, 24], suggesting that CD44 may contribute to the efficiency of distant metastasis through its capacity to function as an adhesion receptor, facilitating the escape of cells from the circulation. Given our prior demonstration that elevated CD44 expression may initiate adhesion of cells to distant endothelial monolayers [23, 24], the objective of this study was to characterize the importance of CD44 in regulating post-intravasation events and distant metastasis of breast cancer *in vivo*.

## RESULTS

### CD44 expression predicts reduced disease free survival and distant recurrence in breast cancer patients

**Figure 1:**
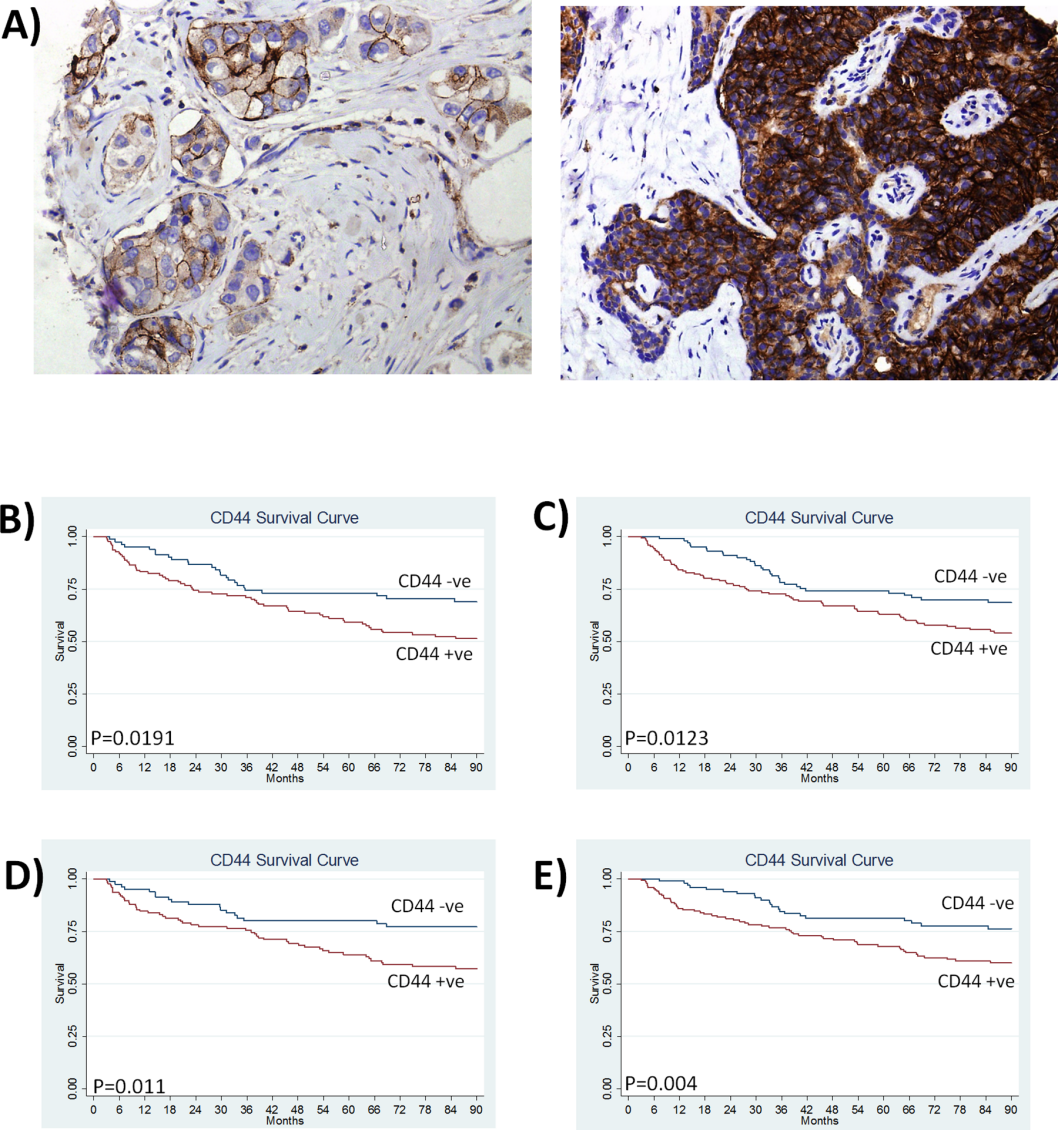
CD44 expression predicts for reduced disease-free survival and increased distant recurrence in breast cancer patients **(A)** Representative images of CD44 immunoreactivity determined by an immuno-histochemical study of tissue microarray sections from a cohort of 448 breast cancer patients. Images shown represent low and high CD44 staining at a magnification of x100. **(B)** Kaplan Meier survival curves stratifying disease-free survival according to CD44 expression in node-positive patients and **(C)** patients with large tumor size (> 2.5 cm). **(D)** Kaplan Meier estimates of distant metastasis-free survival in node positive patients and **(E)** patients with large tumor size (> 2.5 cm) (where recurrence is defined by distant recurrence only).

The clinical significance of CD44 was evaluated by immunohistochemical analysis of a breast cancer tissue microarray constructed using primary tissue from 448 patients. CD44 was predominantly expressed on the plasma membrane of breast tumor epithelial cells (Fig. 1A). Based on the proportion of positive tumor cells and the intensity of the staining, 62% of cases were considered strongly positive for CD44 (Table 1). Strong CD44 expression associated with high-grade tumors (0 = 0.046), progesterone receptor negative (*p* = 0.029) and estrogen receptor α-negative tumors (*p* = 0.001) (Table 1). There was no association of CD44 with nodal status, age or HER2 expression (Table 1).

**Table 1:** Table showing the association of CD44 expression with clinical pathological parameters in a breast cancer patient cohort

Association of CD44 expression with clinical pathological parameters using Fishers exact test				
		%	CD44 %	P
CD44	+		62.5	
	−		37.5	
Grade	I-II	54.3	55.3	
	III	45.7	65.0	0.046
Node	+	51.8	61.5	
	−	48.2	63.6	0.693
Age	> 55	51.5	63.8	
	< 55	48.5	61.1	0.56
ER	+	67.9	57.3	
	−	32.1	73.2	0.001
PR	+	51.5	66.4	
	−	48.5	71.2	0.029
Her2	+	19.5	55.2	
	−	80.5	61.9	0.715

NOTE: Expression of CD44 is expressed as a % of patients in individual clinical classifications (1st column)

Kaplan-Meier analysis confirmed that high CD44 expression predicted for reduced disease-free survival in lymph node-positive patients (*p* = 0.019) (Fig. 1B) and patients with large tumor size (> 2.5 cm) (*p* = 0.012) (Fig. 1C). CD44 correlated with clinically/pathologically-confirmed distant recurrence in the entire cohort (*p* = 0.046) (see Supplemental Figure S1); moreover, the significance value further increased when distant recurrence was considered only in the context of lymph-node positive tumors (*p* = 0.011; Fig. 1D) and large tumors (*p* = 0.004; Fig. 1E).

### CD44 expression is associated with metastasis-related phenotypes

**Figure 2:**
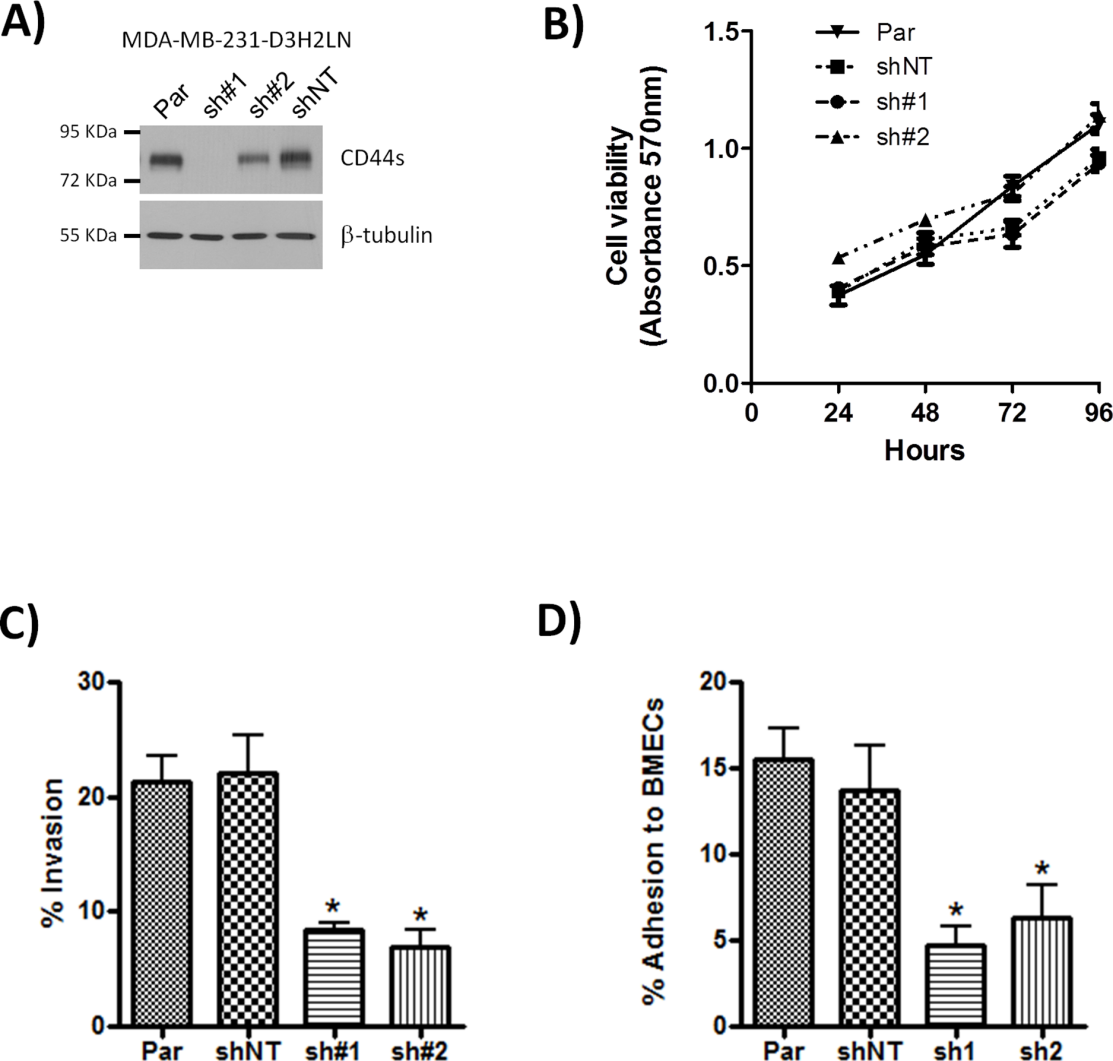
Knock-down of CD44 has no effect on cell proliferation but reduces adhesion and cell invasion **(A)** Western blot showing CD44s expression in parental (Par) MDA-MB-231 cells and following transfection of these cells with either CD44 sh#1, CD44 sh#2 or non-targeting (shNT) control shRNA constructs. **(B)** Curve confirming the absence of an effect of CD44 knock-down on cell proliferation rates, measured using MTT assays performed over 96 hours. **(C)** Bar graph presenting data obtained from modified Boyden chamber assays measuring cell invasion of the indicated clones through Matrigel^®^. **(D)** Bar graph presenting adhesion assay data demonstrating the effect of CD44 knock-down upon the efficiency of adhesion over 30 min of the clonal MDA-MB-231 cells to a monolayer of bone marrow endothelial cells (BMECs). All data shown are mean ± SEM of at least 3 independent experiments (*p < 0.05 by *t*-test)

To ascertain the functional importance of CD44 in the context of estrogen receptor negative breast cancers, we used the CD44-expressing MDA-MB-231 cell line, which retains the CD44^+^/CD24^−/low^ phenotype characteristic of tumor-initiating breast cancer cells. To assist *in vivo* experimentation, we exploited two independent shRNA sequences to repress CD44 expression in luciferase-labeled, MDA-MB-231-Luc-D3H2LN cells. Cells transfected with sh#1 exhibited negligible CD44 expression while transfection with sh#2 resulted in an intermediate level of CD44 repression relative to parental cells and transfection with a non-targeting sequence (shNT) (Fig. 2A). To determine the functional role of CD44 expression we performed a series of *in vitro* assays using parental, shNT-, sh#1- and sh#2-transfected cells. CD44 depletion did not affect cell proliferation (Fig. 2B), nor cause detachment of cells or induce anoikis. However, sh#1- and sh#2-transfected cells were significantly less invasive through Matrigel™ than the parental or shNT-transfected cells (each *p* < 0.05; Fig. 2C) and showed reduced adhesion to a monolayer of bone marrow endothelial cells (BMECs) (*p* < 0.05; Fig. 2D). These functional assays confirm an important role for CD44 in regulating cell adhesion and invasion but not proliferation.

### CD44 expression correlates with increased metastasis and decreased survival to ethically mandated euthanasia in mice

To directly test whether CD44 is required for the post-intravasation stages of the metastatic cascade, tumor formation was assessed following the intra-cardiac injection of shNT-, sh#1-, sh#2-transfected MDA-MB-231-Luc-D3H2LN cells in athymic nude mice. By injecting cells directly into the circulation, this approach eliminates the effects that CD44 may have during the early stages of metastasis and permits an exclusive study of the relevance of CD44 to post-intravasation events. Systemic dissemination and tumor growth profiles of the inoculated cells were monitored *in vivo* using bioluminescent imaging, and confirmed by post-mortem histology.

**Figure 3:**
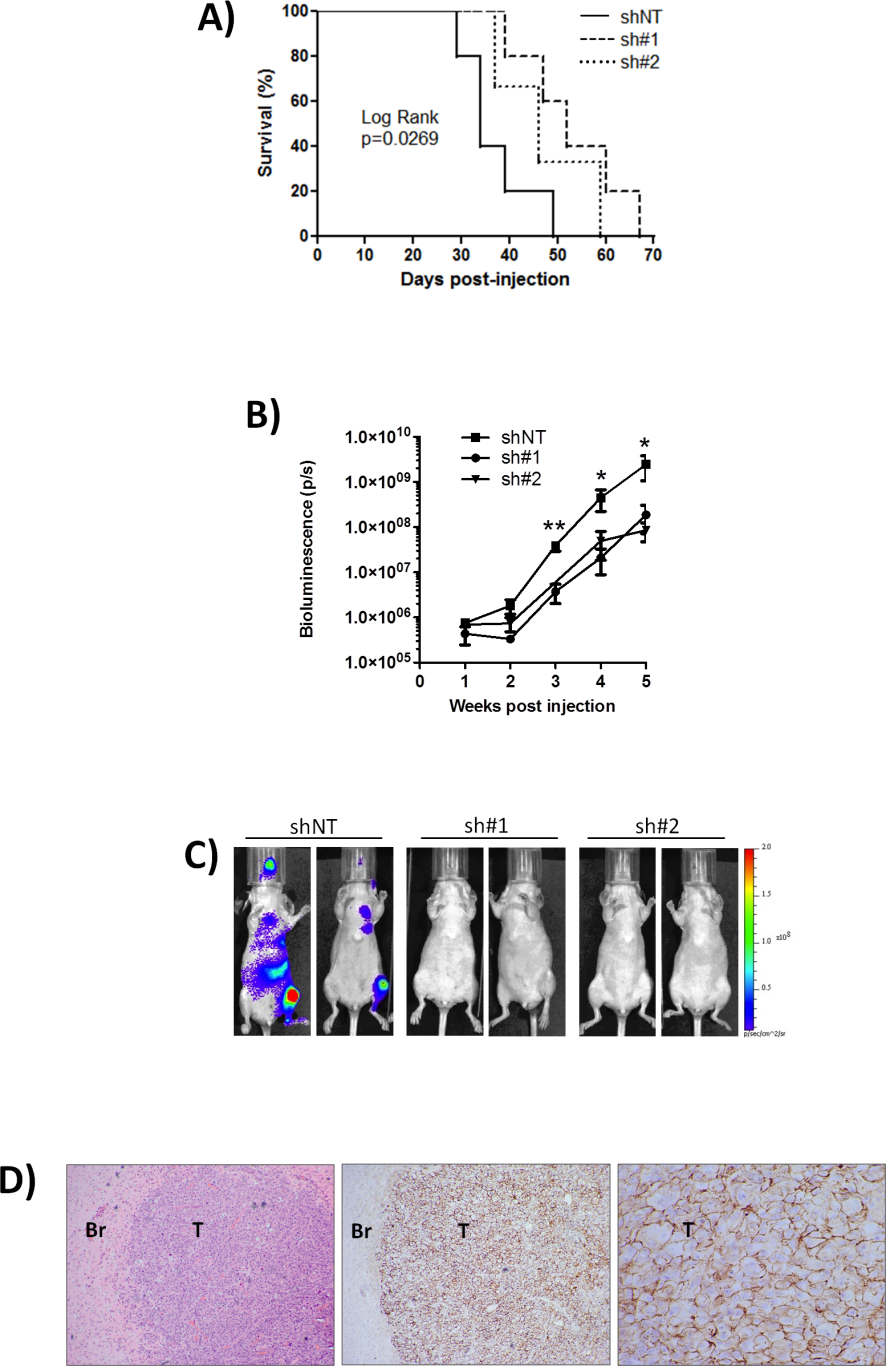
Knock-down of CD44 improves survival to ethically mandated euthanasia and decreases metastasis *in vivo* **(A)** Kaplan-Meier survival curve of mice inoculated with MDA-MB-231 shNT (*n* = 5), CD44 sh#1 (*n* = 5) or sh#2 (*n* = 3) cells (comparison of shNT and sh#1 *p* = 0.0269 by Log Rank test). **(B)** Normalized quantification of bioluminescent signals determined at weekly intervals in mice from shNT (*n* = 7), sh#1 (*n* = 8) and sh#2 (*n* = 7) groups. Sh#1 was significantly different from shNT at weeks 3, 4 and 5 post-tumor cell injection, while sh#2 was significantly different from shNT at week 5 only. Data represent the mean ± SEM (**p < 0.01, *p < 0.05 by *t*-test). **(C)** Representative images of mice from each group taken 35 days post-intra-cardiac injection of MDA-MB-231 clones; multiple metastases are detected in CD44+ve shNT cohort compared to CD44-depleted sh#1 and sh#2 cohorts. Pseudocolour scale bar is consistent for all images. **(D)** Representative H&E stain of a brain section from a mouse inoculated with MDA-MB-231 shNT cells and corresponding tumor-specific CD44 immunohistochemistry. Magnification is x40 for H&E image, x40 for left image of CD44 immunoreactivity and x200 for right image of CD44 staining. *T* = Tumor, Br = Brain.

CD44 knock-down in the injected MDA-MB-231-Luc-D3H2LN cells significantly extended the survival to ethically mandated euthanasia of recipient mice, with the maximum repression of CD44 (using sh#1) increasing median survival from 34 to 52 days (*p* = 0.027) (Fig. 3A). Overall tumor burden was reduced following injection with sh#1- and sh#2-transfected cells relative to control shNT-transfected cells (Fig. 3B). Sh#1 was significantly different from shNT at weeks 3, 4 and 5 post-tumor cell injection. While sh#2 although showing the same trend as sh#1 was significantly different from shNT at week 5 only (**p < 0.01, *p < 0.05). Tumors arising following the injection of shNT-transfected MDA-MB-231 cells were detectable in brain, liver, heart, lungs and bones (Fig. 3C). Comparison on the same bioluminescence scale clearly illustrates the more efficient development of multiple tumors following injection of CD44 positive cells (shNT) compared to animals injected with CD44 depleted (sh#1 and sh#2) cells. Although tumors are also present in the animals injected with CD44-depleted cells the luminescent light emission is below the minimum of the displayed scale in this Fig. 3C. Tumor burden as indicated by bioluminescent imaging was subsequently confirmed by histology. Representative image of CD44 positive brain metastasis arising following injection of shNT cells is shown in Fig. 3D. Notably although CD44 knockdown varies between the two shRNA constructs, there is no significant difference in the tumourigenicity of cells transfected with CD44 sh#1 and sh#2 suggesting that even an incomplete reduction of CD44 is associated with a less aggressive phenotype.

### CD44 expression promotes bone metastasis

**Figure 4:**
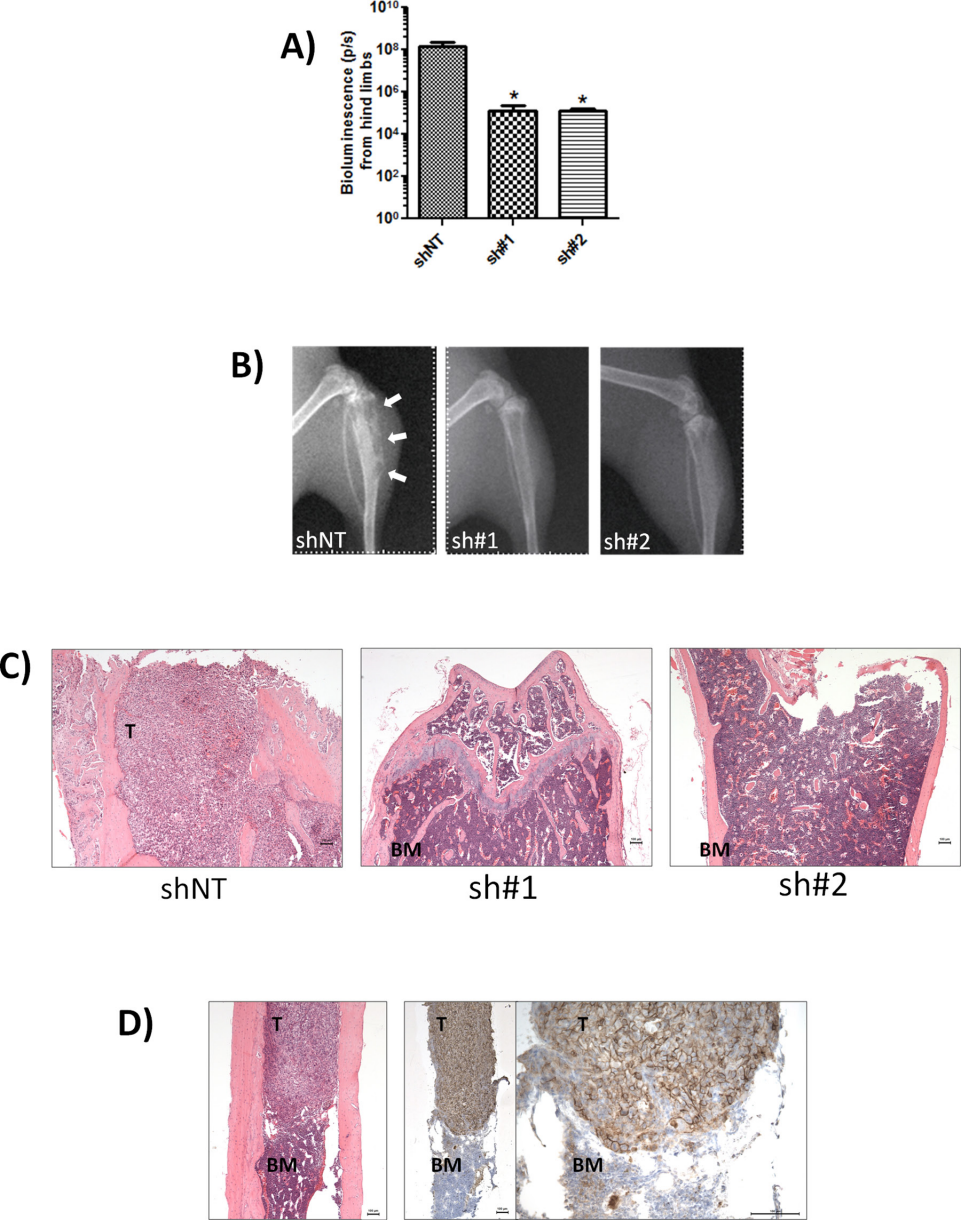
CD44 expression promotes bone metastasis **(A)** Normalised quantification of bioluminescent signals from mice hind limbs injected with indicated clones. Data represents the mean ± SEM (*n* = 6; *p < 0.05 by *t*-test). **(B)** Representative digital radiographs of hindlimbs from mice in each experimental group. White arrows indicate osteolytic bone lesions. **(C)** Representative demineralized bone sections from each experimental group. H&E-stained sections showing complete replacement of bone marrow by tumor in shNT-mice (left) compared with absence of tumor cells in mice injected with sh#1 and sh#2 clones (middle and left). All images are x50 magnification with scale bar equal to 100 μm. **(D)** Representative tumor-specific CD44 immunoreactivity (note strong membraneous staining) and corresponding H&E stain of demineralized bone sections from mice inoculated with MDA-MB-231 shNT cells. Magnification is x50 for H&E image (left) and left image of CD44 immunoreactivity and x200 for right image of CD44 staining; scale bar equal to 100 mm. T = tumor BM = bone marrow.

A high level of metastasis to the hind limbs was clearly evident. Subsequent analysis of hind limbs revealed a reduced tumor burden 4-weeks post-injection in CD44-depleted conditions, with a 1132-fold lower signal detected in the sh#1-transfected model (*n* = 6, *p* = 0.033) and 1137-fold decrease observed in the sh#2-transfected model (*n* = 6, *p* = 0.047) relative to the shNT control (Fig. 4A). X-ray analysis confirmed the presence of osteolytic lesions in the tibia of mice injected with the shNT-transfected MDA-MB-231 cells. In contrast, bone resorption was not apparent in the hind limbs of mice injected with either of the CD44-depleted MDA-MB-231 cells (Fig. 4B). The presence of bone metastasis was confirmed by histology. H&E stained de-mineralized bone sections revealed a decreased tumor burden in the bones of animals injected with sh#1- or sh#2-transfected MDA-MB-231 cells (Fig. 4C). Immunohistochemistry confirmed that bone metastases arising from injection of shNT-transfected MDA-MB-231 cells were strongly positive for CD44 (Fig. 4D).

### Bone-tropic breast cancer cells have elevated CD44 expression and demonstrate increased adhesion properties

**Figure 5:**
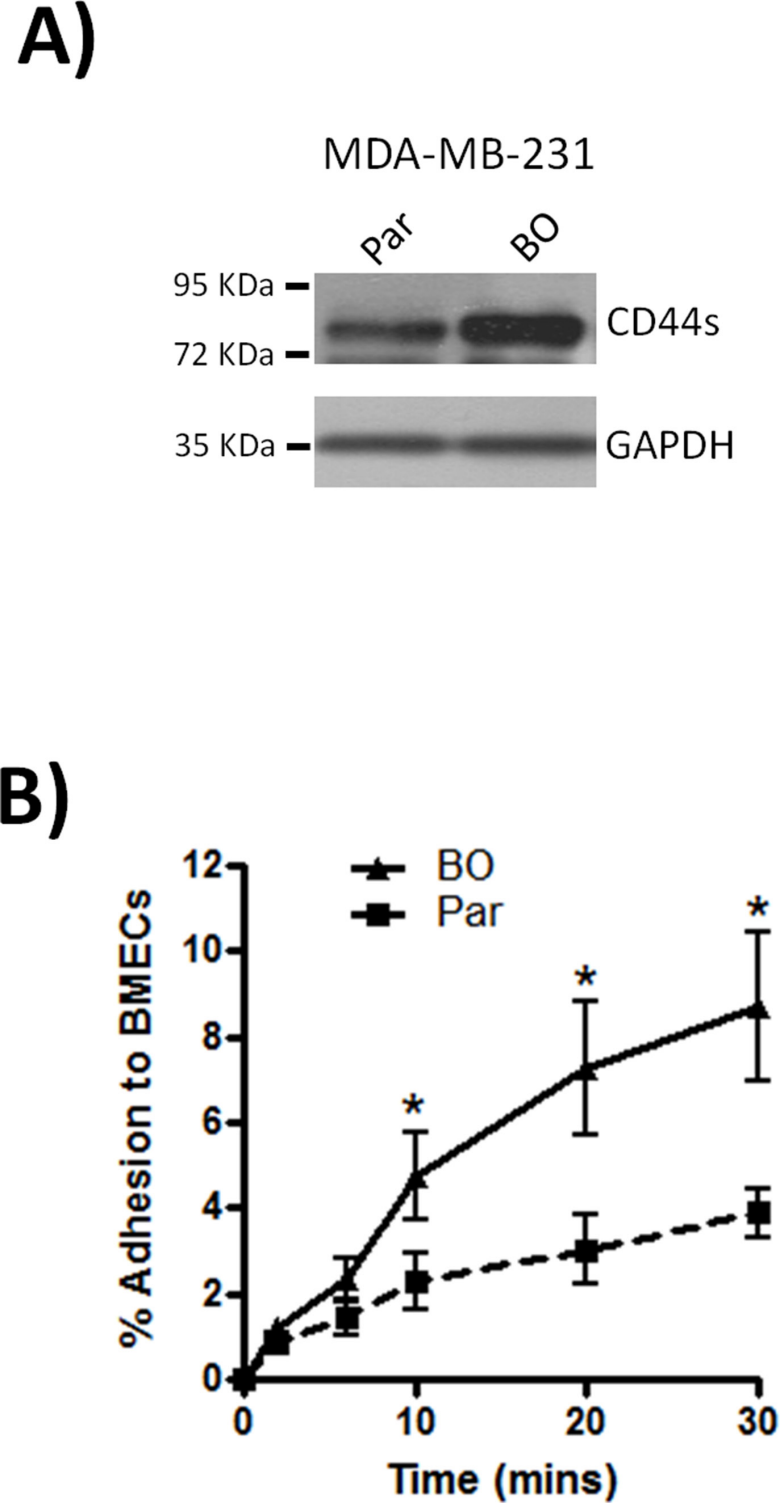
Bone metastatic MDA-MB-231 cells express high levels of CD44 **(A)** Western blot comparing the expression of CD44s between parental (Par) and bone-tropic (BO) MDA-MB-231 cells. Blots shown are representative of 3 independent experiments. **(B)** Comparison of the rate of parental (Par) and bone-tropic (BO) MDA-MB-231 cell adhesion to BMECs over 30 min. All data is the mean ± S.E.M of three at least three independent experiments (*p < 0.05; by *t*-test).

Although metastasis of shNT-transfected MDA-MB-231 cells to bone was not an exclusive event, our *in vivo* study supported a role for CD44 in the efficient outgrowth of metastatic bone lesions. The importance of CD44 in supporting the dissemination of breast cancer cells to the bone was further investigated *in vitro*. Consistent with our *in vivo* results bone-tropic MDA-MB-231 cells (MDA-MB-231BO) expressed higher levels of the standard form of CD44 (CD44s) than the parental cell line (Fig. 5A). Moreover, these bone-tropic MDA-MB-231BO cells were significantly more adherent to BMECs than parental cells at all time points, (*p < 0.05) (Fig. 5B).

## DISCUSSION

Contrary to the findings of several previous immunohistochemistry studies, our analysis of an extensive breast cancer tissue cohort of 448 patients reveals CD44 expression to correlate with reduced disease-free survival and distant metastasis in lymph node-positive patients and patients with large tumor size. In trying to reconcile the obvious differences with published findings, the antibody used in our tissue study detects both standard and variant isoforms of CD44, and therefore, may explain the contrasting results which have correlated CD44s expression, detected by a monoclonal antibody, with favorable overall survival, albeit in a significantly smaller tumor cohort (51 patients) [9]. Furthermore, it is possible that a high CD44v/CD44s ratio in our tumor cohort may also bias the results of our study in favor of poor clinical outcome, consistent with the correlation of CD44v6 with TNM stage [10]. However, it is important to stress that this current analysis has reaffirmed our correlation of CD44 to ER- and PR-null tumors, linking high CD44 expression to basal-like breast cancer, a subtype of this disease known to have the worst prognosis and most aggressive form of disease. As such, the association of CD44 to reduced disease-free survival and distant metastasis in lymph node-positive tumors may not be surprising. Importantly, correlations of CD44s or CD44v expression against these well-established and validated markers in breast cancer were not reported in prior immunohistochemical studies.

Without dispute is evidence in breast cancer that CD44 is expressed by primary tumors, in regional lymph node metastases, and early disseminated “stem-like” tumor cells within the bone marrow of patients [5, 12]. Experimentally, induction of CD44 increases the incidence of MCF-7 cell metastasis to the liver [7]. However, these studies cannot discriminate between the role of CD44 in promoting the initial escape of tumor cells at the primary site from the role it may play in promoting colonization and outgrowth of secondary lesions at distant sites. In focusing on the later stages of the metastatic cascade, our study definitively shows that CD44 contributes to post-intravasation events of the metastatic cascade *in vivo*, where CD44 expression correlates with increased tumor burden at distant sites and reduces survival. This experimental observation reaffirms our correlation of CD44-positivity in primary breast cancer tissue with reduced disease-free survival in patients with confirmed distant metastasis. Integrating this experimental and clinical data therefore provides important and novel insights into the role and importance of CD44 in enhancing the efficiency of later stages of tumor dissemination.

We observed tumor development in multiple tissues and organs following intra-cardiac injection of CD44-expressing MDA-MB-231 cells. Down-regulation of CD44 decreased the rate of onset and the magnitude of tumor burden in the animals, at all clinically-relevant sites including the brain, lung, liver and skeleton, indicating that CD44 does not preferentially select for the metastasis of breast cancer cells to specific tissues. Furthermore, loss of CD44 correlated with an increased survival of the mice. In tissue, CD44 was not statistically associated with disease-free survival across the entire cohort but was significant to disease-free survival and distant metastasis when assessed in the context of lymph-node positive tumors and/or large tumors. Therefore, our studies support that elevated CD44 expression can predict an increased risk of distant metastasis and reduced disease-free survival in discrete sub-populations of breast cancer patients.

*In vivo* modeling confirmed that injection of CD44-expressing cells resulted in the formation of metastatic lesions at multiple, clinically-relevant sites. Bone metastasis is particularly common in breast cancer [25] while the bone marrow may serve as an important site for dormant breast tumor cells [26]. Interestingly, CD44 positive, “stem-like” breast cancer cells have been detected within the bone marrow during early stage disease [3, 5]. Although our data indicates that CD44 does not preferentially select for tissue-specific metastasis *in vivo*, CD44 knockdown on stem-cell-like CD44^+^/CD24^−/low^ MDA-MB-231 cells did significantly decrease tumor burden and reduced the incidence of metastatic lesions in the hind limbs of animals. The importance of CD44 in promoting the colonization of bone by breast tumor cells is further supported by our *in vitro* experiments. For example, we show that bone-tropic MDA-MB-231 cells show elevated expression of CD44 relative to their parental line *in vitro*, and consistent with our previous characterization of the importance of CD44 in initiating cell-cell adhesion [23, 24], these bone tropic cells were more efficient in adhering to BMECs. Thus, elevated CD44 expression on tumourigenic cells may increase the efficiency with which circulating cells can acquire entry to and colonize the bone.

The knockdown of CD44 on MDA-MB-231 cells also decreased their invasion through an experimental matrix. In a further recent study, we have shown that the activation of CD44 results in an increased transcription and synthesis of key proteases of the serine proteinase, cysteine cathepsin and matrix metalloproteinase families [27]. In particular, the capacity of CD44 to increase cathepsin K and MT1-MMP is especially relevant given that these two enzymes are extremely potent in degrading collagen I, and therefore initiate the degradation of the collagen-enriched bone matrix. Therefore, in the context of bone metastasis alone, we propose that the increased level of osteolytic lesions detected in the hind limbs of mice injected with CD44-expressing MDA-MB-231 cells may relate to the capacity of CD44 to not only increase uptake of circulating tumorigenic cells, but also to assist the capacity of these infiltrating cells to initiate turnover of the bone and colonization.

In conclusion, our study confirms that the expression of CD44 increases the efficiency with which tumourigenic breast cancer cells can successfully complete the distant steps of the metastatic cascade. Studies conducted on human tissue and through *in vivo* modeling confirm that CD44 expression correlates with distant metastasis and reduced survival. These observations are consistent with our prior characterization of CD44 in initiating the arrest and adhesion of breast cancer cells on vascular endothelial cells [23, 24] and in upregulating the expression of proteases associated with the degradation of key matrix proteins in clinically-relevant tissues [27]. Overall, our data implies that CD44 can promote tumor cell adhesion to endothelial barriers and facilitating localized invasion/colonization within the tissue, rather than promoting or increasing the rate of cell proliferation at secondary sites. Interestingly, our results take on added clinical significance when one considers that this study and other recent reports indicate that CD44 expression is especially enriched in ER-negative, PR-negative and/or Her2-negative breast cancers which have the worst clinical prognosis and outcome [28].

## MATERIALS AND METHODS

### Antibodies and reagents

Anti-CD44 was obtained from R&D systems, UK. HRP-conjugated secondary antibodies were obtained from Amersham, UK and Hyaluronan (MW220 kDa) from Lifecore Biomedical (MN, USA). Cells were stimulated with 100 μg/ml HA for indicated times. All other reagents were purchased from Sigma (Poole, UK) unless otherwise stated.

### Cell culture

Luciferase-expressing MDA-MB-231 cells (MDA-MB-231-Luc-D3H2LN) were purchased from Caliper Life Sciences (Cheshire, UK). SureSilencing™ CD44-shRNA#1, CD44-shRNA#2 or non-targeting-shRNA constructs (Superarray Bioscience, MD, USA) were transfected using Lipofectamine2000, (Invitrogen, UK).

Puromycin-resistant clones were screened for CD44-knockdown by Western-blotting. A bone-tropic clone of the MDA-MB-231 cell line (MDA-MB-231BO) was provided by Prof. Yoneda, University of Osaka [29]. Cells were maintained in DMEM supplemented with 10%(v/v) FCS (PAA). Human bone marrow endothelial cells (BMECs) obtained from Dr. Weksler (Weill College of Medicine, Cornell University, NY) were maintained in DMEM supplemented with 5%(v/v) FCS, 10 mM HEPES and 3 mM L-glutamine.

### Breast cancer xenograft and bioluminescence analysis

Female athymic nu/nu mice aged 6–8 weeks (Harlan) were anesthetized by inhalation of 2–3% Isofluorane before injecting 1 × 10^5^ sh#1, sh#2 or shNT transfected MDA-MB-231-Luc-D3H2LN cells into the left cardiac ventricle. Bioluminescent imaging was achieved by intraperitoneal injection of D-luciferin (150 mg/kg). Successful intracardiac injection was indicated by systemic bioluminescence throughout the animal; only mice with evidence of successful injection were included in the study. Development of metastasis was assessed by weekly imaging. Metastasis was quantified by selecting a region of interest and measuring the total photon flux (p/s) using Living Image^®^ software v3.2 (Caliper Life Sciences, Cheshire, UK). Background bioluminescence was subtracted from the totals. Animals were weighed twice per week and were euthanized when they lost 20% of their starting weight or when they exhibited first signs of morbidity or paralysis. If any animal developed tumors at sites that would compromise feeding or excretion, they were removed from the study and euthanized by an approved schedule 1 means as stipulated by the Home Office.

### Bone histology

Bones were collected upon euthanasia, fixed in 10% formalin for 24 h, demineralised in 5% EDTA containing 1% formalin for 2 weeks, then processed using standard histological techniques. Demineralised bone sections were stained with Haematoxylin and Eosin or with anti-CD44 (1:400, detection using Envision™ detection kit, Dako).

### Invasion and adhesion assay

Cell invasion and adhesion were assayed as described [23, 24] except tumor cells were harvested by scraping for adhesion assays.

### Patients

Breast tumor samples were collected from archival cases at St Vincent's University Hospital, Dublin, Ireland as previously described [30]. As detailed in table 1 45.7% of cases were high grade tumours, 51.8% of individuals were lymph node positive, 51.5% of individuals were greater than 55 years of age, 60.4% of tumours were greater than 2.5 cm in size, 67.9% were estrogen receptor positive, 51.5% were progesterone receptor positive and 19.5% were Her2 positive. 40.3% of patients received chemotherapy, 60.9% received radiotherapy and 70.1% received tamoxifen.

### Tissue microarray construction and immunohistochemistry

Four μm sections were cut from the TMA and immunostained with anti-CD44 (1:200) on an automated platform (Bond™ system - Vision BioSystems™, Mount Waverley, Victoria, Australia). Sections were counterstained with hematoxylin. Negative controls were included by omission of primary antibody. Positive control tissue (normal thymus) was also used. Immunostained slides were scored on the proportion of positive tumor cells (range 0–5) and average staining intensity of positive cells (range 0–3) which were added together to obtain a total score (range 0–8) as described [31]. Cases were classified as negative (0–2), low (3–5) or high expression (6–8). All cores were examined by two independent observers (SMcF and PT) and 10% were also scored by a third independent observer (EK).

### SDS-PAGE and western-blotting

Cell lysates (20 μg) were separated by SDS-PAGE and transferred onto immobilon-P PVDF membranes (Millipore, Billerica, MA, USA). Membranes were blocked for 1 h at RT in Tris-buffered saline/0.1% Tween-20 (TBS-T) containing 5%(v/v) dried milk. Incubation with primary antibody was carried out at 4°C overnight. Membranes were washed three times in TBS-T and then incubated for 1 h with the appropriate horse-radish peroxidase (HRP)-labeled secondary antibody. Following three washes in TBS-T, immunoreactivity was detected using SuperSignal West Pico Chemiluminescent Substrate (Pierce Biotechnology, Rockford, IL, USA) followed by exposure to X-ray film.

### Statistical analysis

Univariate analysis of tissue-derived data was performed using Fisher's exact test for categorical values and Wilcoxon's test for continuous variables. Multivariate analysis was conducted using Cox's proportional hazard model. A two-tailed *P* value < 0.05 was considered significant. Kaplan-Meier survival analysis was generated and Wilcoxon tests employed to determine significance. For analysis of functional *in vitro* assays and *in vivo* experiments, significance was assessed using Student's *t*-test, two tailed for functional assays and one tailed for analysis of *in vivo* experiments. A *p*-value < 0.05 was considered significant.

### Ethical approvals

Animal studies were approved by the Biological Resource Unit Review Board at QUB and conducted under a Home Office Licence in compliance with UK legislation. Written and informed consent was received from all patients prior to the inclusion of tissue specimens in the TMA on which this study was conducted.

## SUPPLEMENTARY FIGURE


