# Supplementary material for: CD44 increases the efficiency of distant metastasis of breast cancer
Source: Oncotarget. 2015 Mar 19;6(13):11465–76. doi: 10.18632/oncotarget.3410 (PMC4484469; doi:10.18632/oncotarget.3410)
Supplement: Supplementary file 1 [file oncotarget-06-11465-s001.pdf]

SUPPLEMENTARY FIGURE

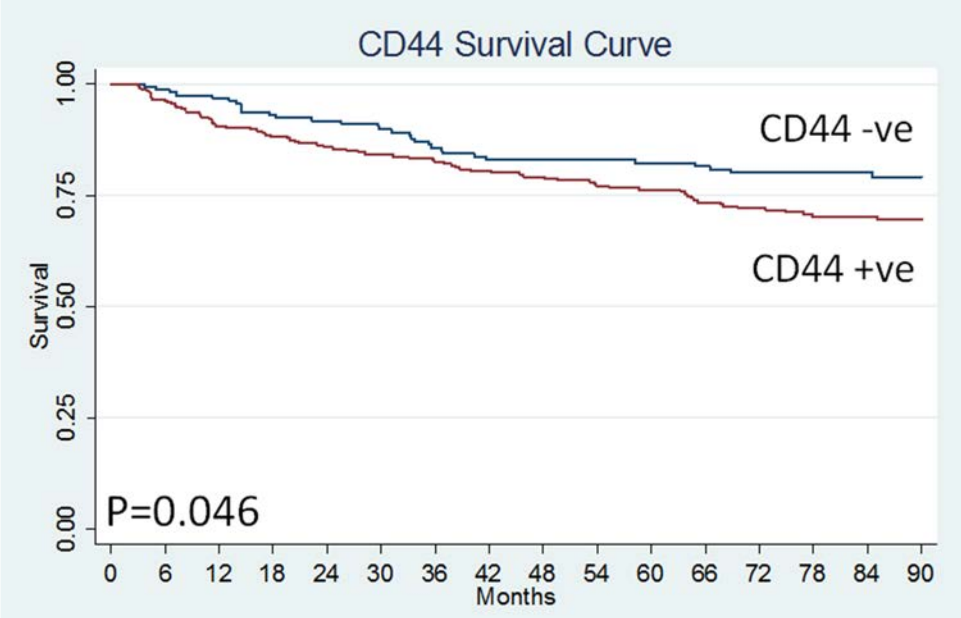

Supplementary Figure S1: CD44 expression predicts increased distant recurrence in breast cancer patients. Kaplan Meier estimates of distant metastasis free survival according to CD44 expression (where recurrence is defined by distant recurrence only).
